# An exploration of trauma-informed care curricula in chiropractic programs: A scoping document analysis protocol

**DOI:** 10.1371/journal.pone.0321498

**Published:** 2025-05-09

**Authors:** Victoria A. Bensel, Zachary Cupler, Olivia Poppen, Jane Joyce, Mike Allgeier, Michael Wiles, Mary Driscoll, Kristy Carbonelli-Cloutier, Brian C. Coleman

**Affiliations:** 1 Department of Biomedical Informatics and Data Science, Yale School of Medicine, New Haven, Connecticut, United States of America; 2 Solomon Research Center at Yale School of Law, New Haven, Connecticut, United States of America; 3 VA Connecticut Healthcare System, West Haven, Connecticut, United States of America; 4 Physical Medicine and Rehabilitative Services, Butler VA Health Care System, Butler, Pennsylvania, United States of America; 5 Institute for Clinical Research Education, University of Pittsburgh, Pittsburgh, Pennsylvania, United States of America; 6 Anesthesia and Physical Medicine Service, VA Orlando Health Care, Orlando, Florida, United States of America; 7 Palmer College of Chiropractic, Davenport, Iowa, United States of America; 8 Northeast College of Health Sciences, Seneca Falls, New York, United States of America; 9 Parker University, Dallas, Texas, United States of America; 10 Surgical Services, VA Pittsburgh Healthcare System, Pittsburgh, Pennsylvania, United States of America; 11 Physical Medicine and Rehabilitation, VA Finger Lakes Health Care System, Rochester, New York, United States of America; 12 School of Health and Rehabilitation Sciences, Doctor of Chiropractic Program, University of Pittsburgh, Pittsburgh, Pennsylvania, United States of America; 13 Universidad Central del Caribe, Bayamon, Puerto Rico, United States of America; 14 Pain Research, Informatics, Multimorbidities, and Education (PRIME) Center, Virginia Connecticut Healthcare System, West Haven, Connecticut, United States of America; 15 Department of Psychiatry, Yale School of Medicine, New Haven, Connecticut, United States of America; 16 Department of Emergency Medicine, Yale School of Medicine, New Haven, Connecticut, United States of America; Iran University of Medical Sciences, IRAN, ISLAMIC REPUBLIC OF

## Abstract

**Background:**

Trauma is a significant public health issue that affects both mental and physical health. Healthcare delivery based on trauma-informed care (TIC) principles is designed to mitigate the risk of re-traumatization in healthcare settings to improve patient outcomes. Chronic pain is a common comorbidity of trauma and a common reason that people seek healthcare, including chiropractic care. The extent to which TIC training is integrated into chiropractic education and Doctor of Chiropractic Programs (DCPs) remains unclear.

**Objective:**

This study aims to evaluate the presence of TIC principles in educational curricula documents from accredited DCPs across the United States and Canada to identify potential gaps in trauma-sensitive education within chiropractic training.

**Methods:**

A scoping document analysis will be conducted using educational curricula documents (program handbooks, course catalogs, and course syllabi) from DCPs accredited by the Council on Chiropractic Education (CCE-USA). Documents will be evaluated for TIC-related search terms based on established frameworks from the Substance Abuse and Mental Health Services Administration and the Harvard Medical School TIC Core Competencies. The analysis will assess the presence of TIC principles such as safety, trust, empowerment, and cultural sensitivity. A phased approach will be used for data extraction, ensuring a comprehensive review of TIC integration.

**Results:**

The study will quantify the inclusion of TIC principles in chiropractic education in the United States and Canada and identify trends or gaps related to TIC education.

**Conclusion:**

Our findings can inform future curriculum review and development, ensuring DCPs integrate TIC effectively to enhance care for trauma-exposed patients.

## Introduction

Trauma is a widespread and significant public health issue that negatively impacts healthcare engagement and outcomes across all demographics. Trauma is defined as events or experiences that are emotionally or physically harmful and that impair a person’s well-being [1–5]. Traumatic events include a wide range of experiences, such as physical trauma, adverse childhood experiences (ACEs), sexual assault, physical or verbal abuse, natural disasters, wartime or combat experiences, witnessing harm inflicted on others, and even profound experiences of loss, stigma, oppression or abandonment [1,5,6]. In the United States, 82.7% of adults surveyed reported experiencing at least one traumatic event in their lifetime [7], and there is a dose-response relationship between the number of traumatic experiences and the risk of mental health issues, chronic pain, disability, and other comorbidities [5].

Given the widespread impact of trauma—psychological, emotional, and physical—across the lifespan, incorporating trauma-informed care (TIC) into healthcare settings is essential. Physical trauma, such as accidents or injuries, significantly increases the risk of developing chronic pain and disability in adulthood, along with emotional and mental health impacts [4–6,8–16]. Adults with a history of ACEs utilize healthcare services more frequently and have over a 25% increase in healthcare expenditures compared to those without a history of trauma [17]. Multiple studies have highlighted the high comorbidity rates between chronic pain and post-traumatic stress disorder (PTSD) [8,18], with up to 10% of people seeking healthcare for chronic pain also experiencing PTSD [8]. Some populations have demonstrated even higher rates of comorbid chronic pain and PTSD, such as children [19] and United States Military Veterans [10,20]. Individuals with both chronic pain and PTSD tend to report more severe pain, higher levels of PTSD symptoms, greater depression and anxiety, more disability, and increased opioid use compared to those with only one of these conditions [8,11–16]. Trauma may complicate chronic pain management and the existing body of evidence underscores the need for specialized, trauma-informed care.

Over and above the health implications of trauma for chronic pain and its management, it is important to recognize that healthcare systems, the providers who serve in them, and the spaces themselves can be triggering for individuals with trauma histories. Rigid expectations and scheduling practices, stigma, hierarchical patient-provider differentials, invasive procedures, medical mistakes, physical exams, staff turnover, forceful recommendations, and chaotic spaces can all be triggering and can interfere with treatment, adherence and trust in persons with a trauma history [21]. Thus, the presence of trauma may be a barrier to equitable and optimal outcomes further reinforcing the importance of a trauma-sensitive system, workforce, and environment.

TIC acknowledges the pervasive effects of trauma and seeks to avoid re-traumatization by embracing six core principles that include safety, trust, peer support, collaboration, empowerment, and cultural sensitivity [1,2] to foster a supportive environment where patients feel both physically and psychologically safe and engaged in their care. The Substance Abuse and Mental Health Services Administration (SAMHSA) emphasizes the importance of TIC to mitigate the long-term health consequences of trauma and to improve patient outcomes [1–3]. Adopting TIC principles in healthcare settings enhances provider confidence and strengthens the therapeutic alliance [22], which is particularly critical in any environment that involves sensitive or potentially triggering procedures.

### Chiropractic care and chronic pain

Acute and chronic musculoskeletal pain are major contributors to global disability and health burdens [22–25]. Clinical practice guidelines for common musculoskeletal pain conditions, such as low back pain, frequently recommend nonpharmacologic approaches as a primary intervention, including manual therapies [24,26–28]. Chiropractic is one clinical discipline that frequently employs manual therapies for the nonpharmacologic management of common musculoskeletal pain conditions [29]. Given their hands-on nature, manual therapies can be sensitive procedures and introduce patient-practitioner power dynamics, particularly related to gender and authority asymmetries [30–32]. Certain manual therapy procedures, such as spinal manipulation, may involve therapeutic touch or patient/practitioner positioning that can make patients feel vulnerable or disempowered. For individuals with a history of trauma, these procedures can lead to re-traumatization, which occurs when a new, similar event triggers stress from a previous trauma, often without the individual recognizing the connection [33–35]. Additionally, providers should be aware of the complex links between post-traumatic stress symptoms and chronic pain outcomes [8,11–15]. Given the high prevalence of chronic pain among individuals with a history of trauma and their likelihood of seeking chiropractic care, it is prudent for chiropractors to be trained in the provision of TIC.

### The gap in chiropractic education

TIC has been introduced into health professions training in geriatrics, nursing, emergency medicine, doula work, osteopathy, dental hygiene, and pharmacy [3,36–41]. However, the inclusion of TIC in chiropractic education has not been explored. This study aims to evaluate the inclusion of TIC principles and training within accredited DCPs across the United States, its territories, and Canada.

## Methods

We conducted a brief, preliminary literature search in PubMed for “trauma-informed care” AND “chiropractor” with search results limited to a single commentary [5], and no results on chiropractic education. Thus, our approach will apply scoping review methods to search educational curricula documents rather than focusing on peer-reviewed literature.

For this review, curricula documents are defined as official materials that outline the educational framework and content for Doctor of Chiropractic (DCP) programs. These documents include program handbooks, course catalogs, syllabi, and course outlines. Curricula documents provide insight into the structure, content, and objectives of chiropractic education and provide an opportunity to appraise the inclusion of TIC principles.

### Eligibility criteria for DCPs

We aim to include all Council on Chiropractic Education (CCE-USA) accredited DCPs in the United States (including Puerto Rico) and Canada (Table 1). Only DCPs with active accreditation status (i.e., Award Initial, Continued, Continued with Warning) awarded by CCE-USA as of November 2024 [42] will be considered eligible for inclusion. DCPs that have discontinued matriculating students or DCPs denied CCE-USA accreditation will be excluded. Additionally, DCPs obtaining active accreditation status (i.e., Initial Accreditation Application, In Process) will also be excluded. Post-graduate programs, such as master’s or diplomate programs offered at DCP institutions, will not be included in this study.

**Table 1:** Eligible Council on Chiropractic Education -Doctor of Chiropractic Programs.

United States Doctor of Chiropractic Program Institution	City, State/Territory
Cleveland University – Kansas City College of Chiropractic	Overland Park, KS
D’Youville College	Buffalo, NY
Keiser University	West Palm Beach, FL
Life University	Marietta, GA
Life Chiropractic College West	Hayward, CA
Logan University	Chesterfield, MO
National University of Health Sciences^*^	Lombard, IL & Seminole, FL
Northeast College of Health Sciences	Seneca Falls, NY
Northwestern Health Sciences University	Bloomington, MN
Palmer College of Chiropractic^*^	Davenport, IA & Port Orange, FL
Parker University	Dallas, TX
Sherman College of Chiropractic	Boiling Springs, SC
Southern California University of Health Sciences	Whittier, CA
Texas Chiropractic College	Pasadena, TX
University of Bridgeport School of Chiropractic	Bridgeport, CT
Universidad Central del Caribe	Bayamon, PR
University of Western States	Portland, OR
**Canada Doctor of Chiropractic Program Institution**	**City, Province**
Canadian Memorial College of Chiropractic	Toronto, Ontario

*Curriculum documents may be standardized across multiple campuses or specific to an individual campus. We will review programs either collectively or separately, depending on the programs operational model in use*.*

### Curricula document collection

We will collect relevant documents directly from the accreditation body and/or individual programs using a two-pass approach. We will first request and aim to obtain relevant records from the CCE-USA for accredited DCPs. Subsequently, we will contact the Academic Dean’s office (or equivalent) for each DCP via email to request additional or updated curricula documents, if available. All information provided for this study will be voluntarily obtained from both CCE-USA and individual DCPs. DCPs will be contacted four times over a one-month period by email before being considered “non-responsive”. For non-responsive DCPs or DCPs who actively decline to share available documents, we will rely solely on materials available through other sources (CCE-USA or publicly available data).

We will request electronically available program handbooks, program curricula, detailed course syllabi, and course descriptions active for the 2024–2025 academic year from eligible DCPs. All documentation must be in English to be considered. Both DCP handbooks and specific DCP courses will be examined for TIC integration across the DCP’s curriculum.

### Incomplete curricula

Incomplete curricula refer to instances where the available documents do not provide a full representation of the DCP’s educational offerings. This could include missing key components such as course descriptions, syllabi, or required courses that are essential for a comprehensive evaluation. For example, if a DCP provides only a general overview without specific course details or omits certain years or sections of the curriculum, we will consider it incomplete. We will distinguish non-responsive programs, incomplete curricula, and complete curricula that lack TIC terminology and training opportunities in reporting results.

### Document analysis framework

Our analysis approach is adapted from SAMHSA’s Concept of Trauma and Guidance for a Trauma-Informed Approach [1] and the Harvard Medical School (HMS) TIC Core Competencies Mapped to HMS Competency Domains [2]. These provide a rigorous, structured framework for identification and evaluation of TIC integration within DCP curricula.

### Selection and vetting of document search terms

The document analysis will use search terms (Table 2) selected based on their direct relevance to the core concepts of TIC as outlined in the SAMHSA and HMS frameworks. These terms were cross-referenced with an interpretive analysis defining the scope of TIC in healthcare settings [43] to confirm their validity as indicators of TIC content in educational curricula. We will adopt direct match and fuzzy match search strategies for these terms, allowing for derivations and variations to be included in our semantic analysis. Our strategy allows for a detailed analysis of the extent to which TIC principles are embedded in each DCP’s curriculum, enabling the identification of specific courses, teaching methods, and assessment strategies related to TIC.

**Table 2:** Key terms for Trauma-Informed Care in Doctor of Chiropractic Programs curricula.

“trauma informed care”	“gender-specific services”	“inquire about abuse”
“informed approach”	“trauma impact”	“self-care strategies”
“sense of safety”	“trauma screening”	“trauma communication”
“psychological safety”	“assessment and treatment”	“patient safety”
“power differentials”	“epidemiology of trauma”	“re-traumatization”
“culturally appropriate”	“human stress response”	“trauma informed approach”
“trauma sensitive”	“trauma”	“empowerment”
“identity and culture”	“resilience”	“safety”
“trustworthiness”	“transparency”	“peer support”
“collaboration”	“mutuality”	

### Ethics

This project was determined to not constitute human subjects research by the Yale Human Research Protection Program (HRPP) and the Department of Veterans Affairs VA Connecticut Healthcare System R&D Committee. This protocol has been registered in the Open Science Framework (DOI 10.17605/OSF.IO/K97P8). All data will be collected and maintained securely with managed access while ensuring that no personally identifiable information is disclosed or misused. The deidentified data that supports the findings of this study will be available upon reasonable request.

### Data screening and extraction

The data screening and extraction process will be conducted in a phased manner, starting with DCP handbooks and course titles and descriptions before progressing to course syllabi. This approach is based on methodologies from previous studies [18,44–46], where search terms were systematically applied to documents to identify specific searchable content within. All review and data extraction procedures will be completed in duplicate to ensure consistency and accuracy.

#### Phase 1: initial screening of program handbooks, course titles, and course descriptions.

The initial screening will involve a review of each DCP program handbook, course titles, and course descriptions. Each program handbook will be searched for TIC-related terms and identified as including or not including features of TIC. Course titles and descriptions will likewise be screened for those that include or suggest the presence of TIC-related terms.

#### Phase 2: screening of course syllabi.

An initial screening will be conducted of the course syllabi to allow us to ensure that any TIC-related content, even if not explicitly indicated in the course title or description, is potentially captured. Any syllabi found to reference TIC-related content will undergo an in-depth analysis to extract relevant details, such as specific course topics related to TIC, course outcome measures, teaching methods (e.g., lectures, workshops, clinical practice), assessment strategies (e.g., examinations, practical assessments), and the integration of TIC principles (e.g., specific topics covered, alignment with SAMHSA’s TIC framework).

### Data extraction

Data extraction will be performed independently by at least two reviewers using a structured extraction form (S1 File) developed based on SAMHSA’s framework and the HMS TIC Core Competencies. The form will capture essential information, including the DCP name and location, course specifics, teaching methods, assessment strategies, and alignment with TIC principles. The extracted reviewer data will be compared to reach consensus, and any discrepancies will be resolved through discussion. If consensus cannot be reached, an independent additional reviewer will adjudicate the differences.

### Data organization and analysis

All data will be collected and organized using a cloud-based, shared spreadsheet to facilitate a descriptive analysis. This approach ensures a systematic and transparent process for identifying and analyzing TIC-related content within DCP curricula across the study team.

### Data analysis and reporting

Study reporting will be informed by and adapted from the PRISMA-ScR recommendations for comprehensiveness and clarity of reporting (S1 Fig). We will report the results of our document analysis using descriptive statistics for quantitative features. We will also conduct a qualitative thematic analysis of TIC integration from identified text data. Identified TIC-related terminology will be tabulated to quantify findings by search term.

### Timeline of study

Data collection began in early to mid-November 2024 by contacting DCPs and the Council on Chiropractic Education (CCE). Our outreach provided an overview of the project and requested access to relevant handbooks, curricula, and TIC-related documents. Initial contacts with DCPs took place between November 8 and November 15, 2024 with up to three follow-ups as needed. By December 2024, collected materials were organized within a secure, shared cloud-based system for team access, followed by preliminary handbook screenings that focused on TIC-specific terminology and frameworks using SAMHSA and HMS guidelines. Detailed data extraction, including consistency checks, continued through late January 2025. Requests for syllabi were made on February 25, 2025, with a timeline for collection and analysis as part of the second phase through April, 2025.

## Discussion

The formal and informal integration of TIC principles within educational curricula is critical for preparing healthcare providers to address the complex needs of individuals who have experienced trauma. This protocol outlines a systematic approach to evaluate the presence and integration of TIC principles in curricula of Doctor of Chiropractic training programs across the United States and Canada, using a combination of phased screening and in-depth extraction processes. By leveraging frameworks from the SAMHSA and the HMS TIC Core Competencies [1,2], this study aims to provide a comprehensive and rigorous analysis of how well TIC is embedded within chiropractic education.

### Significance of the study

Chiropractors frequently encounter patients who have experienced trauma. Given the physical nature of chiropractic care, which often involves direct patient contact and body positioning, there may be inherent risk of re-traumatization if care is not provided within a trauma-informed framework. This study’s findings will be critical in identifying potential gaps in current chiropractic education and highlighting areas where TIC principles can be better integrated. Ultimately, the goal is to inform curriculum development to ensure the incorporation of TIC in chiropractic programs, which may lead to increased patient-centeredness of care, improved patient outcomes, and enhanced practitioner-patient relationships.

### Methodological strengths

The phased approach to data collection, screening, and extraction is a significant strength of this study. By initially soliciting curricula documents based on CCE-USA-provided information and then soliciting DCP’s for institutional documents, this study design will maximize the likelihood of up-to-date, comprehensive data collection. Further, the phased screening and extraction processes ensure a stepwise, thorough, and systematic evaluation of DCPs integration of TIC across their curricula. The use of a standardized form for data extraction, along with independent reviews by multiple reviewers, adds to the rigor and reliability of the findings. The simultaneous search and extraction process also ensures that the study is both efficient and comprehensive in capturing relevant TIC content across a wide range of educational materials.

### Potential challenges and limitations

Despite its strengths, this study may face several challenges. One potential limitation is the reliance on the availability, accuracy, and quality of curricula provided by CCE–USA and the DCPs. If DCPs are unable or unwilling to provide the most recent or complete curricula documents, the analysis may misrepresent the state and extent of TIC integration within those DCPs. Additionally, reliance on specific search terms, while necessary for systematic analysis and slightly mitigated by the use of fuzzy matching, may miss nuances in how TIC is incorporated into curricula, particularly if institutions use different terminology or approaches to cover TIC-related concepts.

Furthermore, the qualitative nature of TIC integration—such as the depth of discussion, the context in which TIC principles are taught, and the practical application of these principles—may not be fully captured through a term-based search and extraction process. Course descriptions and syllabi may not effectively convey every condition or case example covered in each course, or inter-student variation in cases during experiential learning in clinical environments. Some students in the clinical training phase of their education may have robust exposure to TIC principles and training based on individual case presentations and advanced training completed by attending clinicians. Future studies could consider incorporating qualitative interviews with curriculum developers, educators, and students to complement the findings of this document-based review.

### Implications for chiropractic education, the profession, and beyond

The study’s findings can significantly impact chiropractic education by providing evidence-based recommendations for curriculum enhancement. If gaps are identified, accrediting bodies and DCPs may be prompted to update meta-competencies and curricula, respectively, to include comprehensive TIC training. Post-graduate training in TIC principles may also be a strategy to support practicing chiropractors who may not have had exposure to TIC in their pre-clinical and clinical education. This study could serve as a model for similar curricular evaluations for other healthcare disciplines, promoting broader adoption of TIC across the healthcare education spectrum.

## Conclusion

This study protocol describes a systematic and preliminary evaluation of TIC integration within DCPs in the United States and Canada. By evaluating the breadth of TIC principles and training embedded within DCP’s current curricula, the study aims to contribute to the ongoing effort to enhance chiropractic education and improve patient care outcomes. The results of this study will not only inform curriculum development within chiropractic programs but can also influence broader educational practices across healthcare disciplines.

## Supporting information

S1 Fig Preferred Reporting Items for Systematic reviews and Meta-Analyses extension for Scoping Reviews (PRISMA-ScR) Checklist. (PDF)

S1 File Data Extraction Template. (XLSX)
